# Nowhere else to turn? building a case for equity of access to VV ECMO in the UK for complex multi-system patients

**DOI:** 10.1186/2197-425X-3-S1-A475

**Published:** 2015-10-01

**Authors:** R Loveridge, S Patel, V Kakar, C Willars, T Hurst, T Best, A Vercueil, J Wendon, G Auzinger

**Affiliations:** King's College Hospital, London, United Kingdom

## Introduction

In the UK, VV ECMO is only commissioned [[1]] for specific patients - the list of perceived contra-indications is long which prevents some patients having access to this rescue technique. Recognising this, the National Peer Review Programme stated that there may be cases *“who might benefit from ECMO support who also require super-specialist services”* - these may lack equity of access to commissioned resources.

## Objectives

To examine the outcomes of moribund patients, with complex multi-system disease, who fall outside of the present commissioning for VV ECMO, or who are unsuitable for transfer due to specialist tertiary needs.

## Methods

All ECLS patients since service inception in late 2012 had data prospectively collated on their severity of illness.

For our VA group we have recently reported an ELSO SMR (eSMR) for quality assurance. For those who were unsuitable for, or refused transfer to, commissioned VV centres, and in whom we initiated VV ECMO, we analysed outcomes and organ failure.

## Results

There were 49 ECLS runs in 45 patients, & 24 runs within the last year. For VA cases including all eCPR, survival to discharge was 48% (eSMR 0.76). There were 17 VV runs in 16 patients. All were moribund by critical care standards, aged 11 - 52, median 29.5. They included 6 with acute liver failure, 7 in receipt of liver transplant, 4 with complex trauma, 1 tracheal obstruction, 1 post-partum cystic fibrosis & 1 post BMT. One was eCPR, 8 were peri-arrest or had already had a recent cardiac arrest. 100 % met the inclusion criteria for VV ECMO, but 56% were receiving a strict neuro-protective regime for TBI, ICH, post cardiac arrest or cerebral oedema, 75% had severe circulatory failure (50% on > 1 mcg/kg/min noradrenaline), 69 % were on CRRT, 44% had liver failure, 100% had a significant risk of bleeding - mean lactate was 7.65 with 44% having a lactate > 10. Overall, 62.5% had weaned and 50% had survived at the time of this report. They should have both recognition and equity of access to NHS (England) resource. There is nowhere else to turn.

## Conclusions

1. King's College Hospital meets the caseload criteria, ELSO guidelines and quality assurance data for a centre undertaking all modes of ECLS

2. In a young moribund group of patients who were unsuitable for VV ECMO in the national centres, our VV ECMO cohort did excellently with 50% surviving even though such patients would normally be denied this critical care rescue technique - particularly in the UK

3. NHSE should work with hospitals outside of the commissioned centres to ensure equity of access and effective outcomes for patients with complex multi-system disease

4. ECMO should form part of the armamentarium of tertiary critical care services, especially for super-specialist populations that are not represented in the commissioned centres - and there are disadvantages - in terms of equity of access - to overly restricting this service
